# Balancing digital and in-person support: parents’ perspectives on delivering childhood obesity treatment

**DOI:** 10.1186/s12889-026-27659-9

**Published:** 2026-05-20

**Authors:** Paulina Nowicka, Emelie Hemming Bäcklin, Karin Nordin, Markus Brissman, Marie Löf, Karin Eli, Anna Ek

**Affiliations:** 1 Department of Food Studies, Nutrition and Dietetics, Uppsala University, Uppsala, Sweden; 2 Division of Pediatrics, Department of Clinical Science, Intervention and Technology, Karolinska Institutet, Stockholm, Sweden; 3 Department of Medicine, Karolinska Institutet, Huddinge, Sweden; 4 Warwick Medical School, University of Warwick, Coventry, UK

**Keywords:** Child, Obesity, Treatment, Parenting, mHealth, Qualitative research, Interviews

## Abstract

**Introduction:**

Digital formats have the potential to enhance access and engagement of obesity treatment programs, yet little is known about how parents experience them. This qualitative study aimed to explore how parents of children aged 2–6 years perceived and experienced the digital delivery of a well-established and evidence-based parent support program (More and Less, ML) as treatment. The study was part of a larger randomized controlled trial (RCT) within the EU-funded STOP project, which compared the digital intervention with standard care. The treatment included a 10-week online version of the ML parent support program, followed by six months of continued support via a smartphone app. The ML program constituted the active treatment, while the follow-up app was introduced afterwards as a supportive tool to help families maintain changes.

**Methods:**

Semi-structured interviews were conducted with 14 parents from 13 families (12 mothers and 2 fathers, mean age 38 years, range 30–47) in Sweden. Nine held a university degree. Their children (9 girls and 4 boys) were aged 2–6 years; 9 had obesity and 4 had overweight. Interviews were recorded, transcribed, and analyzed using thematic analysis guided by the COM-B model, which focuses on the capability, opportunity, and motivation required for behavior change.

**Results:**

Two main themes were identified: *Supporting each other* (with subthemes *Group support* and *Group learning*) and *Support through digital programs* (with subthemes *Digital vs in-person engagement* and *Staying on track*). Parents highlighted the value of connecting with peers during group sessions, which fostered a sense of community and emotional safety. Although many preferred in-person meetings, the digital format was appreciated for its convenience. Motivation to use the app as follow-up varied; some found it helpful, while others expressed a desire for a more interactive and personalized format.

**Conclusion:**

Parents managing childhood obesity benefit from supportive, nonjudgmental environments. Peer support—even when delivered digitally—enhances their confidence and engagement. Digital tools may also serve as a valuable complement to group-based treatment by extending support beyond the sessions. These findings suggest that future interventions should combine flexible digital components with peer support to enhance engagement and sustainability.

**Supplementary Information:**

The online version contains supplementary material available at 10.1186/s12889-026-27659-9.

## Background

The global prevalence of overweight and obesity among children continues to rise (WHO 2021). In Sweden, approximately 80,000 children are currently living with obesity (Swedish National Board of Health and Welfare 2023). National surveillance data indicate that the prevalence of overweight and obesity among Swedish 4-year-olds increased from 11% in 2018 to 13% in 2020 during the COVID-19 pandemic (Miregård et al. 2023), likely due to disrupted routines, reduced physical activity, and increased food intake during lockdowns. However, the most recent national data from 2022 (Nylander et al. 2025) show a return to pre-pandemic levels, with a prevalence of 11.4%, suggesting that the pandemic-related increase was transient. Despite this decline, the same study found that children in socioeconomically vulnerable households—including those with a single parent or low family income—continue to be disproportionately affected.

In childhood, excess adiposity can initiate a cascade of adverse health outcomes, including early-onset depression, cardiometabolic disease, and chronic inflammation, which may increase susceptibility to conditions such as type 1 diabetes and multiple sclerosis (Marcus et al. 2022). Obesity also contributes to systemic inflammation and significantly elevates the risk of premature mortality. For instance, individuals with childhood obesity face a threefold higher risk of death before age 30 compared to their peers (Lindberg et al. 2020). Importantly, timely intervention before puberty can mitigate these risks. This underscores the need for early treatment strategies that support long-term health outcomes (Marcus et al. 2022; Putri et al. 2025).

Against this backdrop, identifying treatment formats that are both accessible and sustainable is essential—particularly for families with limited access to in-person care. The COVID-19 pandemic highlighted the vulnerability of traditional healthcare delivery, disrupting access to services and reinforcing the importance of digital alternatives. In response, healthcare systems rapidly adopted mobile health (mHealth) technologies—defined as the use of mobile and wireless technologies to support health objectives (WHO 2019)—to maintain continuity of care. In the Stockholm region alone, mHealth app usage increased tenfold during the pandemic (Region Stockholm, 2020), also influencing how childhood obesity interventions were delivered.

Beyond the pandemic context, digital interventions may also offer more enduring benefits by improving accessibility and flexibility of care (Azevedo et al., 2022; Bonvicini et al. 2022). For example, digital delivery can facilitate participation for families living outside urban areas or for those facing practical or socioeconomic barriers to attending in-person sessions. As such, digital formats may have the potential to improve equity in access to childhood obesity treatment.

One such intervention is the More and Less (ML) parent program, a family-based treatment developed for children aged 2–6 years with overweight or obesity. It is grounded in Social Learning Theory, Social Interaction Theory, and Bronfenbrenner’s ecological systems theory, which together highlight how parenting practices, social context, and broader environmental factors shape children’s health behaviors (Ek et al. 2015; Bandura 1971; Bronfenbrenner 1992). Previous trials have demonstrated that ML is more effective than standard care for weight reduction in young children, particularly when supplemented with additional support (Ek et al. 2019a). In Sweden, standard care typically involves follow-up within child health care or outpatient pediatric services, with support from healthcare professionals such as pediatricians and nurses, and when needed, referrals to dietitians, psychologists, or physiotherapists. In 2019, the program was included in the EU-funded ML Europe study, implemented in Sweden, Romania, and Spain (Ek et al. 2019b). The ML program was originally intended for in-person delivery, but during the COVID-19 pandemic in 2020 it was rapidly adapted to an online format to ensure continuity of access for participating families.

Sustaining behavior change after initial treatment remains a key challenge in childhood obesity interventions. Follow-up support has been shown to play an important role in maintaining treatment effects and supporting long-term behavior change. Previous research has shown that follow-up booster support delivered after the core group-based ML program contributes to more sustained treatment outcomes (Ek et al 2019a, b; Ek et al 2023). However, it remains unclear how such follow-up support can be delivered in alternative formats, including digital modalities, and how parents perceive and experience digital support in relation to in-person and group-based care.

This study aimed to evaluate parents’ experiences and perceptions of the ML parent program delivered digitally, and how they experienced the subsequent follow-up support provided through a smartphone app. While digital health solutions are increasingly used in pediatric obesity treatment, there is limited qualitative research exploring how parents perceive both group-based treatment and digital follow-up support—particularly for preschool-aged children. This study addresses this gap and contributes to a deeper understanding of how families engage with digital formats in real-world settings. By focusing on parents’ perspectives, this study also provides insights that may inform the design and implementation of more accessible, acceptable, and sustainable interventions, particularly in relation to fostering engagement and maintaining participation.

## Methods

### Participants

This qualitative study was conducted in the Swedish arm of the ML Europe study, with 2–6-year-old children with overweight or obesity (Ek et al. 2019b). ML Europe was an EU-financed randomized controlled trial conducted in Romania, Spain, and Sweden (n = 100 per country), aiming to treat overweight and obesity in preschoolers. Children were randomized 1:1 into either a control or an intervention group (ML program). The control group received standard treatment as defined by national guidelines. In Sweden, this followed the Stockholm County guidelines Action Plan for Overweight and Obesity 2016–2020, involving 4–6 appointments (30 min each) over one year, including visits to a dietitian, physiotherapist, and pediatrician (Region Stockholm 2016). For this study, we conducted semi-structured interviews with Swedish parents of children with overweight or obesity who participated in the intervention group.

### Intervention

The ML program is a well-established and evidence-based obesity treatment program (Ek et al 2019a, b, 2023). ML was originally designed for in-person delivery, but due to restrictions during the COVID-19 pandemic it was adapted to a digital format. Sessions were delivered by health care professionals via Microsoft Teams in weekly 1.5 h meetings over ten weeks. Each session introduced evidence-based parenting skills—such as encouragement, limit setting, monitoring, problem-solving, or positive involvement—and covered topics related to food and physical activity (Ek et al. 2019a, b). This provided us with the opportunity to evaluate parents’ perceptions of the digital delivery of the ML program. Furthermore, to evaluate whether incorporating digital follow-up support could replicate the previously observed benefits, we also included a digital follow-up component. For this purpose, we chose to include the MINISTOP app (developed by co-author ML). The MINISTOP app is a six-month universal program to promote healthy lifestyle behaviors in preschool-aged children (Delisle et al. 2015), which has undergone ten years of research from effectiveness to implementation aspects. The app has previously shown beneficial effects on lifestyle behaviors such as decreased screen time and lower intakes of sweetened beverages, as well as high usage and satisfaction among parents and increased parental self-efficacy to improve lifestyle behaviors (Alexandrou 2023a; Alexandrou 2023b). Thus, upon completing the ML program, parents were offered access to the MINISTOP app for six months.

The app functioned as follow-up support and was intended to help families maintain healthy routines established during the ML sessions. It was available for both iOS and Android systems, and parents received an introduction to the app at the time of access, with individual technical support provided when needed. The app included an extensive program with push notifications and information on healthy eating and cooking, physical activity, and sedentary behavior (in total 12 themes delivered every other week). In the app, parents were also encouraged to register their child’s daily intake of fruits and vegetables, sugar-sweetened beverages and sweets, as well as physical activity and screen time at their own preference. Personalized feedback was then provided as push notifications based on the parents’ reports. See Supplementary Material 1 (Table S1) for a summary of the themes included in the app.

### Recruitment

Purposive sampling was used to include parents who had fully completed the digital ML program and subsequently accessed the MINISTOP app, in order to gain in-depth insights into experiences of both the digital group sessions and the follow-up app. This strategy ensured a rigorous and comprehensive understanding of parental experiences (Babbie 2020).

Eligible participants were parents from families (n = 21) who had completed the full digital ML program and downloaded the MINISTOP app between 2020 and 2022. Families could be represented by either one parent or both parents.

All 21 families were contacted by telephone by a member of the research team, following a letter of invitation in November 2022 sent by the ML Europe research group. One family declined due to personal reasons; one father became ill and was unable to participate; six families did not respond to follow-up attempts. In total, 14 parents agreed to participate. Interviews were scheduled at their convenience between 16 November 2022 and 25 January 2023. Interviews took place approximately five to 18 months after participants completed the nine-month intervention. Interview timing depended on when individual families had participated in the ML Europe study.

### Interviews

Data were collected through semi-structured interviews, allowing flexibility for participants to share their perspectives freely, thereby enriching the data (Järvinen & Mik-Meyer 2020). The interview guide contained 13 open-ended questions designed to explore parental perceptions of the digital ML program and the MINISTOP app (see Supplementary Material 2). It was developed specifically for this study by EHB in consultation with the research team, informed by the study aims, prior research on the ML program, and relevant theoretical perspectives on behavior change; it has not been previously published.

Probing techniques were used to elicit detailed responses, including both scripted and spontaneous follow-up questions to guide the conversation (Willis 1999). One pilot interview was conducted with a parent participant, resulting in the addition of a probe: *Would you have used the app* *more frequently if it had been introduced at a different time*?

All participants were asked the same core questions, for example:

- What motivated you to participate in the program?
- How did you experience the digital group format and interaction with other parents?
- What are your thoughts on the MINISTOP application? How did you use it?

Depending on their answers, follow-up questions such as *Can you give an example?* or *How did that affect you?* were used.

Interviews were conducted by EHB via telephone, either from her home or from the university. Parents participated from their homes. Interviews lasted 29–50 min (mean ~ 40 min). No prior relationship existed between the interviewer and participants. Thirteen interviews were conducted in Swedish, and one in English. No field notes were taken, and transcripts were not returned to participants.

### Data analysis

Thematic analysis was conducted following Braun and Clarke’s six-phase approach to thematic analysis (Braun & Clarke 2006). When applying this approach, we were mindful of their later conceptualisation of reflexive thematic analysis (Braun & Clarke 2019). In practice, this meant that the analysis was treated as an interpretative process, informed by the research team’s disciplinary backgrounds and theoretical commitments, with theme development subject to continuous querying and refinement across the co-authorship team.

To deepen the analysis and clarify mechanisms behind behavior change, the COM-B model (Capability, Opportunity, Motivation—Behavior), developed by Michie et al. (2011), was applied after theme development to structure and interpret how parents described factors influencing behavior change, and to guide the discussion of the findings. According to the model, behavior change results from interactions between three key elements:

- Capability: Physical and psychological capacity to adopt new behaviors.
- Opportunity: External influences such as social and environmental supports.
- Motivation: Internal processes including emotion, habit, and decision-making.

These elements are interrelated. For instance, increased capability or opportunity can strengthen motivation. The COM-B framework was used to interpret which factors parents perceived as enabling or hindering behavior change in their families.

EHB transcribed all interviews verbatim and reviewed transcripts thoroughly. Initial coding was inductive, supported by NVivo 12 (v.1.7.1), using both semantic and latent codes to capture explicit and underlying meanings. Codes were grouped into themes and subthemes. EHB conducted the initial coding of all transcripts. To enhance analytical rigor, coding and theme development were discussed iteratively within the research team. KN reviewed and provided feedback on the coding process, and themes were further developed through an ongoing dialogue with AE and PN. This collaborative process supported reflexive interpretation and ensured that multiple perspectives informed the analysis. In line with reflexive thematic analysis, coding and theme development were understood as an interpretative process rather than a search for consensus or reliability. No predetermined codes were used.

The research team consisted of academic and clinical professionals with backgrounds in dietetics, pediatrics, physiotherapy, and qualitative research. These disciplinary perspectives informed the analytical process, including interpretation of the data and development of themes. Throughout the analysis, reflexive discussions were held within the team to consider how researchers’ prior experiences and assumptions may have shaped the interpretation of the findings, including how themes were developed, particularly in relation to understanding parental experiences of digital and group-based interventions.

The study is reported in accordance with the COREQ (Consolidated Criteria for Reporting Qualitative Research) checklist (see Supplementary Material 3).

### Descriptive statistics

Descriptive statistics were used to report participants’ age, sex, and education level. Means (M), standard deviations (SD), frequencies (n), and percentages (%) were calculated using SPSS version 28 (IBM Corp.).

### Informed consent and ethical considerations

All participants signed informed consent upon entry into the ML Europe study. Ethical approval was obtained from the Regional Ethics Committee in Stockholm (dnr: 2018/2082–31/1, approved December 11, 2018); thus, the study adhered to the Declaration of Helsinki.

Participants were informed about confidentiality, the study’s purpose, and their right to withdraw. Verbal consent was audio-recorded before each interview. Participant data were de-identified, and only research team members had access to the material. Given the sensitive nature of the topic, interviewer EHB, a female and experienced district nurse, was well-prepared to minimize risk and ensure participant comfort throughout the interviews. Participants did not receive any financial compensation for participation.

## Results

Fourteen parents from 13 families were interviewed: 86% (n = 12) mothers and 14% (n = 2) fathers, with a mean age of 38 years (range 30–47). Eight mothers had a university degree, and of the two interviewed fathers one had a university degree. The mean age of the children at baseline was 4.2 years, with 69% (n = 9) girls and 31% (n = 4) boys. Thirty-one percent had overweight (n = 4), and 69% had obesity (n = 9).

For context, in the broader ML Europe intervention population (n = 64), 64% (n = 36) of mothers and 48% (n = 29) of fathers had a university degree. The mean age of the children was 4.8 years, with 63% (n = 40) girls and 37% (n = 24) boys. Twenty-eight percent (n = 18) had overweight, and 72% (n = 46) had obesity.

**Fig. 1:**
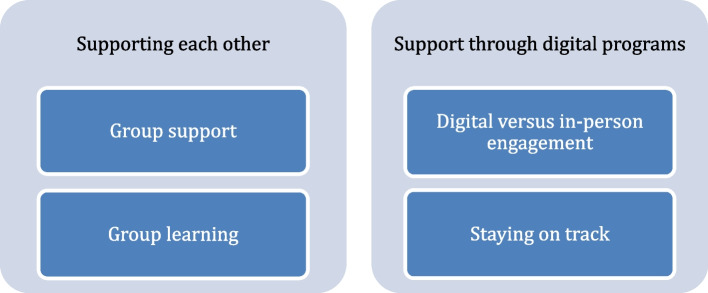
Themes and subthemes

Two main themes were identified, with two subsequent subthemes each. The first theme, *Supporting each other*, consisted of the sub-themes *Group support* and *Group learning*. The second theme, *Support through digital programs*, included the subthemes *Digital versus in-person engagement* and *Staying on track*. See Fig. 1. Together, these themes illustrate how social and contextual factors shaped parents’ opportunities and motivation to engage in behavior change.

### Supporting each other

This theme refers to the peer-based emotional and informational support that parents received during the ML group sessions. It encompasses the mutual encouragement, shared experiences, and practical guidance exchanged between participants. The subthemes *Group support* and *Group learning* reflect how this sense of community contributed not only to emotional relief but also to stronger engagement with the treatment and greater confidence in making lifestyle changes for their children.

#### Group support

Parents reported that the group sessions provided them with an opportunity to share experiences and insights with others facing similar challenges regarding their child's weight. All parents expressed a strong desire for support and a safe space to share their thoughts and concerns, and they found this support within the group sessions. They reported feeling relieved to be able to discuss their struggles and everyday life with others who understood what they were going through. The sessions provided the parents with a valuable platform to connect, share, and learn from each other.

... it was so lovely to hear that others also have the same problem and to hear some other perspectives and things like that … I was just so pleased ... I was almost touched every time … It was so nice … listening and hearing what the others said, and asking questions and getting relevant answers … I think it was really wonderful. (Parent no. 1)

Many parents felt lonely and guilty over their child's weight issues. As a result, some reported losing trust in their parenting capability. The group sessions helped reduce these feelings through new parenting strategies and perspectives on responding to their children's wants and needs to support a healthy weight status.

... it was nice to know that there are others who also struggle in life ... As a parent, one may feel a significant amount of blame, or one may burden oneself with guilt … it was good to know that others also have a hard time ... (Parent no. 6)

The group sessions offered parents a safe and supportive space to explore sensitive topics, such as how to address their child’s emerging self-image and protect them from being seen—or seeing themselves—as different because of their weight. These conversations strengthened and empowered parents, boosting their confidence in supporting their child’s self-perception in a positive and constructive way.

I'm not afraid to talk about things that may be difficult and challenging. That's where my improvement and development and everything took off … I have been strengthened along the way and gained a different mindset. (Parent no. 8)

As this quote shows, through engaging with the group, parents felt empowered to speak with their children about overweight and obesity while maintaining a supportive approach.

Some parents felt that the discussions around self-esteem and self-confidence were among the most important parts of the program and believed they should be given even more space. These topics—often raised and explored within the peer group—were seen as essential to children’s emotional well-being and development. Hearing how other parents navigated sensitive conversations and supported their children’s confidence helped participants reflect on their own knowledge gaps. They valued learning practical, real-life strategies from peers who had faced similar challenges, and felt reassured that they were not alone in their uncertainties. Through these exchanges, parents identified areas where they lacked tools to fully support their children’s self-image and expressed a desire for further guidance on how to build confidence and self-acceptance at home.

This focus on the whole family often stemmed from parents’ concerns about the potential psychological impact of the treatment on their child. Because the children did not attend the sessions, parents could introduce lifestyle changes in a positive, non-stigmatizing way—without drawing attention to weight or framing it as a problem. Many expressed deep relief that their child’s self-esteem remained intact throughout the process.

… it has been a concern for me from the beginning that [daughter’s name] will be marked as weighing too much ... That the excessive weight should be seen as a problem instead of this being an opportunity to think a little more about how we can be physically active and what we eat ... (Parent no. 5).

... She does not need to become aware of these things [having overweight/obesity], it was really important and comforting for us to get help at such an early age before she even realized that she might have problems with these things [overweight or obesity]. So, therefore, we didn't want to make a big deal out of it at all. (Parent no. 1)

A few parents expressed concern about the program ending, worrying about how they would manage without the continued support of the group and its leaders. One parent described how the parent group had helped them accept their situation and cultivate greater compassion and self-acceptance as a parent. This parent realized that their child's weight was not solely their responsibility but shaped by broader influences. The group sessions gave them hope and courage to foster healthier lives for their children.

... it feels a little sad that it's over ... but at the same time ... now we are ready to stand on our own feet … we have probably changed our way of looking at our situation. (Parent no. 5)

#### Group learning

Parents reported that the group sessions became an arena where they exchanged experiences and provided each other with guidance and support. Some parents emphasized how fellow participants helped one another find answers to difficult questions and develop solutions by sharing tips. Although the parents received help and guidance from the group leaders, they felt it was even more important to learn from other parents. Through peer-learning, parents found that making lifestyle changes became easier and that their mindset around their child’s weight began to shift.

The group sessions became a place where parents could discuss how to improve as caregivers, reflect on both mistakes and successes, and—regardless of their parenting strategies—identify things they could be proud of.

... to come so close to other families and see how they act and react ... I think it's useful … to feel proud and strengthened in what one does well and what one thinks is right … (Parent no. 5)

Parents found value in connecting with others facing similar challenges and appreciated the wisdom and support they received. In addition, these interactions gave parents new tools and skills that they could use to make positive changes in their homes and in their children’s lives.

... I hadn't thought I would gain much from talking to other parents like that. That part ... surprised me about how much ... wisdom there is and how, together in some way, [we] can contribute with different perspectives and angles and tips and tricks. (Parent no. 7)

Through sharing experiences, some parents realized that changing behaviors and lifestyles was not as complex as they had initially thought. For some, participating in the parent group became the push they needed to make significant lifestyle changes.

Although some parents noted that not all of their initial expectations of the parent program were fully met, they reflected on how the group leaders played a key role in facilitating learning and reflection. Through supportive language and structured use of the manual, the leaders helped parents connect general recommendations to their own family routines. As one parent described:

The group leaders' commitment was absolutely fantastic ... you feel like, yes, we're not alone in this, and this is going to be great ... it was just a positive experience for me ... and the way we were received has been really nice. (Parent no. 1)

Even when the content itself was familiar, the group discussions enabled parents to view their challenges from new perspectives. These conversations created opportunities for shared interpretation, helped normalize uncertainty, and prompted practical takeaways. Several parents described how they continued to use the manual’s tools at home—adopting strategies like positive reinforcement and encouragement—as a way of reinforcing what they had learned in the group sessions.

All parents described how participation in the sessions prompted them to reflect on their own parenting strategies and beliefs. Engaging in dialogue with others helped them re-evaluate everyday routines and recognize areas where they wanted to grow. For some, these conversations illuminated knowledge gaps they hadn’t previously identified. One parent reflected on the value of having access to such information and a forum earlier in their parenting journey:

This type of information should be available earlier ... to capture all the parents who feel this way, as we are not experts and don't have complete knowledge about it [parenting, nutrition, and physical activity]. It would have been helpful for us from the beginning. (Parent no. 13).

This highlights a desire among parents for earlier and more sustained opportunities for collaborative learning around healthy routines and parenting.

### Support through digital programs

This theme describes how parents experienced and perceived the digital format of the ML sessions and how it influenced their participation in the group (Digital versus in-person engagement). It also covers parents’ perceptions of the MINISTOP app as follow-up support for weight management (Staying on track).

#### Digital versus in-person engagement

All parents reported that they found the digital format of the group sessions convenient. It enabled them to participate more easily in the sessions, particularly given their busy lives, full-time work, and occasionally sick children. For some, digital solutions were essential in allowing them to take part in the program. Moreover, most parents were already familiar with digital meetings due to the pandemic, as described by Parent no. 1:

For me, it was absolutely wonderful [digital meetings], and since I work a lot on Teams all day, it was completely okay ... I don't think it becomes impersonal; it's what you make of it. … I almost think that I might have been more uncomfortable sitting together in a physical group”. (Parent no. 1)

For me, being able to participate [digital meetings] was a requirement for it even to happen, so I value it more than the social aspect and discussions that can arise, but of course, it is a limitation as well [not meeting other parents physically]. (Parent no. 3)

Although most parents appreciated digital meetings, others felt that the digital setting hindered open communication. Three parents described the digital format as tense and noted that it inhibited discussion. Technical issues further complicated participation for some, making it more difficult for everyone to engage actively.

It [discussions and relationships] becomes more detached when you meet digitally … one may not choose to open up in the same way or make yourself heard … it becomes easier just to sit passively. (Parent no. 7)

Another parent, whose first language was not Swedish, found that the language barrier was a greater challenge in the digital setting, making active participation more difficult.

The parents reported missing valuable social interactions that they believed would have been easier to foster in in-person meetings. Many expressed a preference for meeting at least once during the program to build stronger bonds and connections, noting that such relationships were difficult to establish through online communication alone. Some parents specifically mentioned missing the informal conversations typically shared during coffee breaks in in-person settings, and the unstructured experience sharing these conversations might foster.

Several parents also expressed a desire to maintain connections with other participants beyond the duration of the program, as one parent described:

On some occasions, it felt like I almost wanted to continue, like wanting to meet some of the families on the side and continue discussing more freely. (Parent no. 9)

Overall, parents agreed that in-person sessions would have significantly enriched their experience and helped them form more meaningful relationships with each other. However, given the time constraints of parenting young children, most still considered the digital format a suitable and practical alternative.

#### Staying on track

The follow-up support provided through the MINISTOP app after the parent program generated mixed experiences among the parents. All parents downloaded the app, but their usage varied. Most reported using it several times during the first three months (out of a six-month period), while three parents continued using it throughout the full duration.

Parents used the app for different purposes. While many focused on the data entry functions, others primarily used the informational content. Several parents found the app helpful in the beginning, as it offered a structured way to monitor their child’s lifestyle habits:

… In the beginning, I thought that it was useful … the biggest benefit was keeping track of things like sugar intake, sweets and physical activity. (Parent no. 3)

However, over time, many parents discontinued their use of the app, finding it repetitive and time-consuming to log the same information daily. They suggested that the app would be more beneficial if its functions were tailored to individual family challenges, allowing for more personalized feedback and tips.

The feeling then was somehow that the [app] … reminded you of everything you should have in mind with exercise, diet, and such things. But it felt like … we were already doing all of that, and then it was like going in and checking something off that we already had been doing. (Parent no. 9)

It would be great if one could set their own goals ... adapted to their own situation and consider what we need to think about. (Parent no. 13)

Another reason why some parents stopped using the MINISTOP app was that they felt they had already gained sufficient knowledge and guidance from the group sessions and the manual, which included strategies for improving nutrition and physical activity. Some parents also found the initial effort required to start using the app too demanding, which led them to abandon it early.

... the threshold [for using the app] became too big for me compared to having this manual (the ML program) and to go back to and look in. That was easier than starting using an app ... but I also think it depended on us feeling that the material in the manual worked so well. (Parent no. 5)

Although most parents found the app helpful for confirming that they were on track with their lifestyle changes, many felt that the content became repetitive over time. Additionally, three parents shared that the app’s feedback had a negative emotional impact. One parent said that the feedback led them to adjust their entries to reflect a healthier routine than was actually the case. Another parent explained that the feedback affected how she viewed herself:

... when she didn't eat enough vegetables and the feedback wasn't green [positive] ... I became sad ... like I haven't done my job ... I felt a little bad when we weren't active enough. We haven't managed, I haven't been a good parent this week. (Parent no. 2)

Four parents described a positive experience with the MINISTOP app. They felt inspired to improve their routines and habits after receiving feedback, whether it was encouraging or constructive, with three parents primarily using the data entry function, and one parent reading content on the app but not entering data. Of note, three of these parents used the app consistently throughout the entire six-month period. They appreciated how the app helped them stay on track in managing their child’s weight and reported that it became a valuable tool in their daily lives.

[The app] … is good for getting a grasp on reality, seeing how things actually are, and getting concrete feedback on when we have succeeded in doing things differently. (Parent no. 12)

I think all parents should have this application, to track behaviors and nutrition. From screen time to what vegetables and fruit they eat, what sugar they eat, what sugary drink they are taking… you know. It was good, very good. (Parent no. 4)

Most parents shared suggestions for how the app could be improved. Some proposed introducing the app during the group sessions, allowing time for discussion, guidance, and reminders. Others suggested that the app be made available after the final study visit (at 24 months from baseline) to help reinforce long-term behavior change. One parent recommended incorporating gamification to make the app more engaging. Another suggested integrating a forum for peer-to-peer conversation with other parents in the group, which could offer continued tips, encouragement, and support—functioning as an extension of the parent program.

## Discussion

This study explored how parents experienced the ML parent support program, delivered in digital format. As a qualitative study, our aim was not to evaluate the effectiveness of the intervention, but to understand how parents experienced and made sense of its components. Two main themes were developed: *Supporting each other* and *Support through digital programs*. Parents valued connecting with others facing similar challenges, describing the group sessions as a safe space for learning, emotional support, and practical advice. Many felt empowered and more confident in managing their child’s health. The digital format of the ML group sessions was convenient and enabled participation, although some missed the deeper social connection that in-person meetings could provide. We also explored how MINISTOP, an app originally developed as an evidence-based universal support program, could serve as valuable support after the ML program. While most parents appreciated the MINISTOP app initially, many stopped using it after a few months, feeling they had already gained sufficient support from the program. Others found the app helpful for tracking habits and staying motivated. Overall, digital group-based support was well received, and a mobile phone-based program may offer valuable follow-up—if tailored to families’ needs. In the following sections, the findings are interpreted using the COM-B model to better understand how parents’ experiences relate to capability, opportunity, and motivation for behavior change.

### Peer support as a driver for change: Capability, motivation, and opportunity in group treatment

Parents are central agents of change in childhood obesity treatment, especially in early childhood when behavior modification largely occurs within the family context (Davison et al. 2013). This study underscores how peer support, embedded in group treatment, fosters psychological capability and reflective motivation. Parents valued the safe, non-judgmental space where they could share challenges, exchange strategies, and feel less alone. This environment enhanced their self-efficacy and helped diminish guilt—a barrier commonly reported in other studies (Haugstvedt et al. 2011; Andreassen et al. 2013). These findings align with the interpersonal benefits described in the ML program follow-up, where parents reported that emotional and social support within the group helped them cope with weight-related stigma and stress (Nowicka et al. 2022).

The shared learning that occurred during the ML sessions empowered parents with both emotional and practical tools, reinforcing their psychological capability. Parents with low initial confidence in handling their child’s weight concerns often left the sessions with a renewed sense of competence. This echoes Yalom’s (1995) concept of “interpersonal learning,” where group interaction provides insight and strength. Furthermore, peer exchanges reduced the sense of isolation and shame—an important motivational factor also described by Sjunnestrand et al. (2024), where parents expressed ambivalence and emotional strain in conversations about their child’s weight and welcomed support from others with similar experiences. These findings align with Sjunnestrand et al. (2025), who found that parents often experienced weight-related discussions in pediatric healthcare as emotionally charged or unanticipated. In contrast, the group-based format of the ML program allowed for shared understanding and gradual trust-building, which parents described as a more supportive context for addressing concerns about their child’s weight.

In the COM-B framework, the group setting itself also represents an opportunity. For many participants, the digital format increased accessibility by removing logistical barriers—consistent with findings by Kulik et al. (2017) and Roberts et al. (2021). However, some parents noted limitations in the depth of connection in digital meetings (further elaborated in Sect. "Digital formats and mHealth as opportunity and challenge: Balancing accessibility, capability, and motivation"). This sentiment reflects previous research suggesting that social closeness and continuity of care from both peers and professionals are essential for long-term engagement (Nowicka et al. 2022). A blended model, combining in-person and digital sessions, may therefore enhance opportunity for change by balancing flexibility with relational depth.

Beyond emotional and motivational support, the program’s structured manual and group dialogue also contributed to parents capability. Together, these elements supported parents in reflecting on and adjusting their everyday routines. These skill-building aspects mirror findings from Nowicka et al. (2021), who describe parents' sustained efforts to manage the home food environment as a dynamic process of "ongoing negotiation."

Importantly, the social dimension of group treatment helped parents reflect on their broader parenting values—not just related to weight, but also to their child’s self-esteem and emotional wellbeing. This was especially crucial given parents’ concerns about reinforcing stigma or negative body image (Toftemo et al., 2013; Ek et al. 2020). Addressing interpersonal dynamics early in treatment may therefore serve as a protective and enabling factor in sustainable behavior change.

### Digital formats and mHealth as opportunity and challenge: Balancing accessibility, capability, and motivation

The digital format of the ML parent program created new opportunities for participation by removing barriers such as travel, time constraints, and childcare. For several parents, the digital set-up was not only convenient—it was essential to their participation in group sessions. These findings support previous research highlighting how logistical factors influence treatment accessibility (Kulik et al. 2017; Roberts et al. 2021). The online group format thus extended the opportunity for behavior change, a key COM-B component.

However, this opportunity was nuanced. While some parents found the digital environment more comfortable and conducive to sharing, others reported that online meetings inhibited deeper conversations and emotional connection. The absence of informal interactions, such as chatting during coffee breaks, made it harder for some to build trust and social cohesion. This echoes recent work by Sjunnestrand et al. (2024), who found that opportunities for peer support and meaningful weight-related dialogue were particularly valued by parents when they occurred in emotionally safe and interpersonal contexts.

Parents also suggested that a hybrid model—starting and ending the program with in-person meetings—might better support group bonding and foster trust. This aligns with findings from the current ML study as well as prior qualitative insights on the role of interpersonal factors in treatment satisfaction and adherence (Nowicka et al. 2021; Nowicka et al. 2022).

Regarding the MINISTOP app, experiences varied. For some parents, the app supported motivation by reinforcing positive behaviors and providing feedback on diet and activity. Other parents described that the app’s repetitive structure and feedback reduced their motivation or, in some cases, triggered feelings of guilt—especially when goals were not met. These results contrast with previous findings about the MINISTOP app where parents have reported high satisfaction, engagement, and support for behavior change (Alexandrou 2023a, Alexandrou 2023b). Parents’ differing experiences with the app may be understood in light of its original design as a universal prevention tool, whereas in this study it was introduced as follow-up support after group-based treatment. The MINISTOP app is intended for use in child health care services in Sweden and is offered to all families, regardless of the child’s weight. The results in this study emphasize the importance of prior co-creation with end-users to address their needs and expectations to reach the full potential of digital tools. Further research is needed to explore what a mobile app-based follow-up (such as the MINISTOP app or another digital tool) might look like in terms of content, timing, and perceived usefulness after a parent group-based support program for childhood obesity, such as the ML group sessions.

Ultimately, digital formats, such as remote group sessions and a mobile app-based program, provided clear opportunities for participation and continuity of support. However, the variability in engagement underscores the importance of matching support formats to family needs, digital literacy, and psychological readiness for change.

Importantly, these findings do not represent a contradiction but rather reflect different dimensions of social interaction. While parents valued the sense of support and shared experience within the structured group sessions, the digital format for the group sessions was perceived as limiting opportunities for more informal and spontaneous social interactions that might foster deeper connections.

### The dynamic interplay of capability, opportunity, and motivation in behavior change

Parents’ psychological capability to engage in the program was strengthened by peer support, knowledge-sharing, and concrete parenting strategies. Many parents gained confidence in navigating difficult situations with their child, particularly around food, routines, and body image—key areas of concern also identified in other studies (Sjunnestrand et al. 2024; Nowicka et al. 2021; Nowicka et al. 2022). The opportunity to hear others’ perspectives helped parents shift their own thinking, moving from blame and guilt to action and acceptance.

Motivation—both reflective and automatic—was central to parents’ willingness to engage in treatment. While some were initially hesitant, concern for their child’s future health and self-esteem became powerful drivers for participation. This internal motivation was amplified by the opportunity to meet others in similar situations, reinforcing a sense of belonging and shared purpose. The group format thus served both as a motivational boost and as a mirror in which parents could reflect on their own values, fears, and goals.

The opportunity offered by the program, to connect with other parents, was enhanced by its digital format. Digital delivery expanded access, allowing parents to join without logistical strain. However, emotional opportunity—particularly the development of deeper interpersonal connections—was sometimes limited by the digital format, as discussed above. As previous research has shown (Nowicka et al. 2022), these interpersonal dynamics are vital not only for engagement but also for sustaining motivation and reducing the emotional burden of weight-related stigma.

The MINISTOP app further exemplified how behavior change is shaped by the interaction of COM-B components. Parents with higher digital fluency and strong motivation tended to use the app more consistently, while for others the app did not provide the support they needed.

Overall, this study demonstrates that behavior change in the context of childhood obesity treatment is not driven by any single factor but by the dynamic interplay between capability, motivation, and opportunity. Group-based support emerged as a particularly powerful facilitator, not only by building knowledge and skills but also by reshaping beliefs and reducing stigma. Digital formats for group sessions were perceived as valuable for accessibility and continuity; although some parents experienced limitations in terms of social connection and engagement. Further research is needed to elucidate how digital follow-up tools after group treatment can be designed and delivered to adequately support families with different needs.

While this study focused on a specific intervention, the findings may be relevant to similar family-based and digitally delivered programs. In particular, our results highlight the importance of peer support, and flexibility in delivery formats, as well as suggests improved features (e.g., personalization) for tailoring digital tools to families’ various needs when used after obesity treatment.

### Limitations

This study has some limitations that warrant consideration. As a qualitative study conducted within a specific intervention and context, the findings are not intended to be generalizable, but may be transferable to similar settings and interventions.

Participants were recruited among parents who had completed the intervention and agreed to be interviewed. This may have resulted in a sample with more favorable experiences compared to those who discontinued participation. Parents who did not complete the program may have had different perspectives, including potential barriers to engagement, which were not captured in this study. Future research should aim to include these perspectives to provide a more comprehensive understanding of participation in digital and group-based interventions. At the same time, focusing on completers enabled in-depth exploration of how parents engaged with and made sense of both the group sessions and the follow-up support.

The sample was relatively homogeneous, with most participants being mothers, native Swedish speakers, and highly educated. This may limit the transferability of the findings, particularly to families with lower educational attainment or non-native speakers. The sample also differed somewhat from the broader ML Europe intervention population, which may further influence the transferability of the findings. However, it reflects the local context of Stockholm, where educational levels are higher than the national average (Statistics Sweden [SCB], 2021, 2022).

In addition, participants’ relatively high educational level and familiarity with digital tools may have influenced their engagement with and perceptions of the digital format. These factors may differ in other populations, particularly among families with lower digital literacy or less experience with digital communication platforms.

Interviews were conducted approximately five to 18 months after participants completed the nine-month intervention, which may have affected participants’ recall of specific intervention components. To support credibility, open-ended questions were used to promote free expression and reduce the risk of social desirability bias. Thematic saturation—defined as the point at which no new themes or insights were identified—was reached after ten interviews, consistent with previous guidance on sample adequacy in qualitative research (Malterud et al. 2016). This suggests that the data were sufficiently rich to support the study’s conclusions.

All interviews were conducted and initially coded by one researcher, which may have influenced data collection and interpretation. However, regular discussions within the research team supported reflexive analysis and helped ensure that multiple perspectives informed the findings.

Finally, the MINISTOP app was originally developed as a primary prevention program for all children in child health care services in Sweden, regardless of body weight, to promote healthy eating and physical activity, and was therefore not specifically designed to be used as a treatment tool following the ML group sessions. In this study, it functioned as follow-up support rather than an active treatment component. As reported above, the app has shown beneficial effects on health behaviors and high satisfaction among parents and health care professionals when provided as a universal support program within child health care services (Alexandrou 2023a; Alexandrou 2023b).

### Implications for practice

The findings of this study have several implications for the design and delivery of childhood obesity interventions. Digital group formats may enhance accessibility and continuity of care, particularly for families who face barriers to attending in-person sessions. However, maintaining opportunities for meaningful peer interaction remains important, suggesting that hybrid or interactive formats may be beneficial. In addition, further research is needed to understand how digital follow-up tools in obesity treatment can be designed to support sustained behavior change, for example through increased personalization. These considerations may support the development of more accessible and effective family-based interventions in clinical and public health settings.

## Conclusions

This qualitative study explored parents’ experiences of a digital format of the ML group-treatment program for preschoolers with overweight or obesity. The findings, including those from delivering the group sessions online, confirm the central role of peer support in enhancing parents’ capability, opportunity, and motivation—core components of behavior change according to the COM-B model—and high satisfaction with the ML program. However, while the digital format for the delivery of the ML group sessions improved accessibility and convenience, many parents missed the deeper social bonding that in-person meetings might offer. Our findings also showed that a digital app-based six-month follow-up after the group sessions was perceived as helpful by some but not all, and may require further tailoring. Altogether, our findings suggest that while digital formats can offer important opportunities for access and continuity, their effectiveness may depend on how well they align with families’ needs, capabilities, and motivational drivers. To support long-term change, future interventions should integrate tailored and socially responsive components—whether through blended delivery formats or more personalized mHealth features—to optimize engagement and sustainability in early childhood obesity treatment.

## Supplementary Information

Supplementary Material 1.

Supplementary Material 2.

Supplementary Material 3.

## Data Availability

The dataset supporting the conclusions of this article are available upon request from the first author.

## Abbreviations

COM-B: Capability, Opportunity, Motivation–Behavior
ML: More and Less
mHealth: Mobile health
RCT: Randomized controlled trial
STOP: Science and Technology in childhood Obesity Policy
