# Supplementary material for: Balancing digital and in-person support: parents’ perspectives on delivering childhood obesity treatment
Source: BMC Public Health. 2026 May 20;26:1711. doi: 10.1186/s12889-026-27659-9 (PMC13214379; doi:10.1186/s12889-026-27659-9)
Supplement: Supplementary file 1 — Supplementary Material 1. [file 12889_2026_27659_MOESM1_ESM.docx]

**Supplementary file 1**

Content of More and Less program and MINISTOP app

Table S1. Session content of the More and Less program and themes included in the MINISTOP app.

| *More and Less Parenting Program* | |
| --- | --- |
| Session | Content |
| 1 | Welcome and overview |
| 2 | Food and play: When more? When less? |
| 3 | Parents as teachers: cooperation and energy balance |
| 4 | Parents as teachers: to teach children new behaviors |
| 5 | Rewards and incentives |
| 6 | Pre-teaching |
| 7 | Parents as teachers: limit setting strategies |
| 8 | Power struggles: to avoid and to handle them |
| 9 | More support – Less stress |
| 10 | Summary: parenting, food and play – to prepare for the future |
| *MINISTOP app* | |
| Theme | Content |
| 1 | Healthy foods in general |
| 2 | Breakfast |
| 3 | Healthy small meals |
| 4 | Physical activity and sedentary behavior |
| 5 | Candy and sweets |
| 6 | Fruits and vegetables |
| 7 | Drinks |
| 8 | Eating between meals |
| 9 | Fast food |
| 10 | Sleep |
| 11 | Foods outside the home |
| 12 | Foods at special occasions |

Session content of the More and Less program and themes included in the MINISTOP app.
