# Supplementary material for: Balancing digital and in-person support: parents’ perspectives on delivering childhood obesity treatment
Source: BMC Public Health. 2026 May 20;26:1711. doi: 10.1186/s12889-026-27659-9 (PMC13214379; doi:10.1186/s12889-026-27659-9)
Supplement: Supplementary file 2 — Supplementary Material 2. [file 12889_2026_27659_MOESM2_ESM.docx]

**Supplementary file 2**

Interview guide

You and your child participated in the More and Less Europe study and were randomly selected to participate in a parent group. After attending the parent support groups, you accessed the MINISTOP app, which was intended as a follow-up support after the group sessions. This interview aims to understand your experiences of the ML program and the MINISTOP app. The interview will be recorded, and some annotations may be made during the interview. The interviews will be treated anonymously and only by those of us who are involved in the study. Furthermore, there are no right or wrong answers to the questions that will be asked, and the interview will last approximately 40 minutes. Do you agree to participate?

Now that some time has passed since the parent group, we are curious about your experience of participating in More and Less during the group meetings and afterwards, but first, we would like to ask some preliminary questions.

Why did you want to participate in the study?

Did you or someone else attend most group sessions?

What were your expectations of the group meetings?

How did the groups meet your expectations?

Did you have the opportunity to talk about what you wanted?

Are there other things that should have been addressed in the groups? If so, what? Examples?

During the COVID pandemic, we had to think differently and have digital groups via Teams instead of physical groups.

How did you experience the digital groups?

What are the advantages of the digital format?

What are the disadvantages of the digital format? Could we have done anything differently to make the digital groups better?

Would you choose to participate in digital or physical meetings?

Why would you choose that?

This treatment was done in a group.

How did you feel about meeting other parents in this way?

How do you think the digital format affected you and your relationship with other parents in the group?

After the parent group meetings, you had access to the MINISTOP app. The app was intended as a follow-up support and reminder of what was discussed during the meetings.

We are curious about what you thought of using the app.

How did you use it?

Was it easy/hard to use?

Did both parents use the app?

Did one parent use it more than the other? Why do you think that was?

What did you think of the app's content?

Was there any part of the app that you thought was good? Recipes (what was good about them)? Texts (anything particularly helpful, what was good about them, how did you use them, can you give an example)? Registration function/data entry (see below)? Messages (push notifications)?

Do you think the app influenced your habits? If so, in what way?

Was there anything you missed? If so, what?

The app had a registration function to register fruits and vegetables, screen time, activity, sweetened drinks, and snacks. So now we have some questions about that registration function.

Did you use the registration feature in the app? If yes: Can you tell me how you used the registration feature?

How did you use it?

Which of the registration parts was most beneficial for you?

How did you experience the feedback on what you registered? If no: Why not?

What would have needed to be different for you to use the app?

Would you have used the app more frequently if you had received it at a different time?

There is much talk in the media and on social media about treatment in healthcare and how healthcare professionals talk about health and weight. Can I ask you some questions about that?

Did you tell your child that you participated in a parent group?

What did you tell your child about it?

What did your child say then?

Do you think your participation has influenced your child in More and Less parent groups?

If yes: In what way?

Did the child's self-image get influenced? i.e. how the child perceives her/himself.

How have you handled it?

If no: Why do you think that is?

Summarizing your participation in the study, how meaningful do you feel the parenting support program and the MINISTOP app have been for your family?

If we were to redo this study, do you have any suggestions for changes we could make?

Do you want to add or change anything before we finish?
